# Synthesis of Nitroxide Diradical Using a New Approach

**DOI:** 10.3390/molecules25112701

**Published:** 2020-06-11

**Authors:** Pavel Fedyushin, Tatyana Rybalova, Nargiz Asanbaeva, Elena Bagryanskaya, Alexey Dmitriev, Nina Gritsan, Maxim Kazantsev, Evgeny Tretyakov

**Affiliations:** 1 N. N. Vorozhtsov Institute of Organic Chemistry, 9 Ac. Lavrentieva Avenue, 630090 Novosibirsk, Russia; feduyshin@nioch.nsc.ru (P.F.); rybalova@nioch.nsc.ru (T.R.); nasanbaeva@nioch.nsc.ru (N.A.); egbagryanskaya@nioch.nsc.ru (E.B.); maximkazantsev1988@gmail.com (M.K.); 2 Department of Physics, Novosibirsk State University, 2 Pirogova Str., 630090 Novosibirsk, Russia; dmitralexey@gmail.com (A.D.); nina.gritsan@gmail.com (N.G.); 3 V. V. Voevodski Institute of Chemical Kinetics and Combustion, 3 Institutskaya Str., 630090 Novosibirsk, Russia; 4 Department of Natural Sciences, Novosibirsk State University, 2 Pirogova Str., 630090 Novosibirsk, Russia

**Keywords:** fluoroarenes, aromatic nucleophilic substitution, *tert*-butylanilines, nitroxides, diradicals

## Abstract

A new synthetic pathway to diradical organic systems is proposed. The effectiveness of this approach was exemplified by the synthesis of a new nitroxide diradical. An interaction of perfluorobiphenyl with lithium *tert*-butylamide, followed by oxidation of the thusly formed *N*4,*N*4′-di-*tert*-butyl-2,2′,3,3′,5,5′,6,6′-octafluorobiphenyl-4,4′-diamine with *meta*-chloroperoxybenzoic acid, led to the polyfluorinated nitroxide diradical, *N*,*N*′-(perfluorobiphenyl-4,4′-diyl)bis(*N*-*tert*-butyl(oxyl)amine), with a good total yield. The polyfluorinated diradical is stable and can be isolated in free form and completely characterized. The structure of the nitroxide diradical was proved by single-crystal X-ray diffraction analysis. According to the X-ray diffraction data, the diradical is considerably twisted: dihedral angles between the planes of the nitroxide groups and aromatic cycles are 65.1° and 69.5°, and between aromatic cycles 52.6°. Quantum chemical calculations predict well-balanced size of both intramolecular and intermolecular exchange interactions with *J* from −2.65 to −1.14 cm^−1^.

## 1. Introduction

Multi-spin organic molecules possess intriguing properties that not only are of basic research interest but also have a significant potential for advanced technological applications [1,2,3,4,5,6,7]. Notably, these multi-spin molecules have recently emerged as promising building blocks for organic magnetic sensors [8], memory devices [9,10,11], spintronics [12], and spin filters [13,14], as well as for the probing of quantum interference effects in molecular conductance [15]. Taking into account the specific chemistry of a particular class of organic radicals, various approaches to the synthesis of multi-spin paramagnets have been developed previously. For example, multi-spin molecules containing the *tert*-butylarylnitroxide spin carrier have been prepared in accordance with the synthetic scheme including an interaction of appropriate aromatic organometallic compounds with 2-methyl-2-nitrosopropane, thereby producing corresponding *tert*-butylhydroxylamines, which are then oxidized to the desired polyradical products [16,17,18,19,20]. The polyradicals thus obtained have been effectively used for the construction of molecular magnets capable of cooperative magnetic ordering at 3.4–46 K [21,22,23,24]. Realizing that the advances in the synthesis of nitroxide multi-spin molecules would be crucial for the further development of advanced organic magnetic materials and devices, we recently developed a new synthetic approach to functionalized *tert*-butylphenylnitroxides via substitution of a fluorine atom in polyfluorinated arenes by *tert*-butylamine with subsequent oxidation of the obtained *tert*-butylanilines with *meta*-chloroperoxybenzoic acid (*m*-CPBA) [25].

Attempts to apply this concept to the synthesis of multi-spin molecules have shown its limitations. The fact is, *tert*-butylamine reacts only with highly activated polyfluorinated compounds, and this state of affairs makes impossible the introduction of two or more amino groups into aromatic substrates. The solution, as we demonstrated in this work, is the use of *tert*-butylamine in activated deprotonated form. The proposed procedure was employed for the first time to synthesize a *tert*-butylphenylnitroxide diradical, namely *N*,*N*′-(perfluorobiphenyl-4,4′-diyl)bis(*N*-*tert*-butyl(oxyl)amine. We successfully isolated the diradical in a pure form and solved its molecular and crystal structures. To the best of our knowledge, this is the first polyfluorinated nitroxide diradical synthesized so far.

## 2. Results and Discussion

**Scheme 1:**
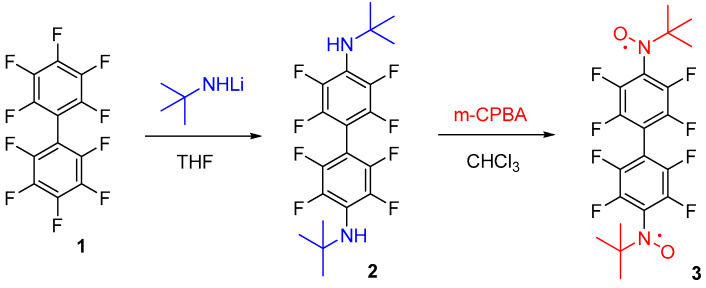
Synthesis of fluorinated nitroxide diradical **3**.

As in the case of *tert*-butylarylnitroxide monoradicals, a synthetic pathway to di- and polyradicals may be the nucleophilic substitution of two or more fluorine atoms in an activated arene under the action of *tert*-butylamine, followed by oxidation of the obtained di- or polyamines into the target multi-spin compounds. We attempted to carry out a reaction of perfluorobiphenyl **1** with *tert*-butylamine, aiming to synthesize the corresponding diradical **3**. Nonetheless, polyfluorinated substrate **1** did not have enough reactivity and the reaction with the amine leading to diamine **2** proceeded slowly. The use of *tert*-butylamine in deprotonated form, namely lithium *tert*-butylamide in tetrahydrofuran (THF), led to the substitution of two fluorine atoms in **1** and the formation of *N*4,*N*4′-di-*tert*-butyl-2,2′,3,3′,5,5′,6,6′-octafluorobiphenyl-4,4′-diamine (**2**) in a yield of ~50%. Formation of other isomeric diamines were not detected. The oxidation of diamine **2** with *m*-CPBA was performed at room temperature and provided target nitroxide diradical **3** as a red crystalline compound in a yield of ~80% (Scheme 1). Diradical **3** was stable and was comprehensively studied both in solution and in the solid state.

**Figure 1:**
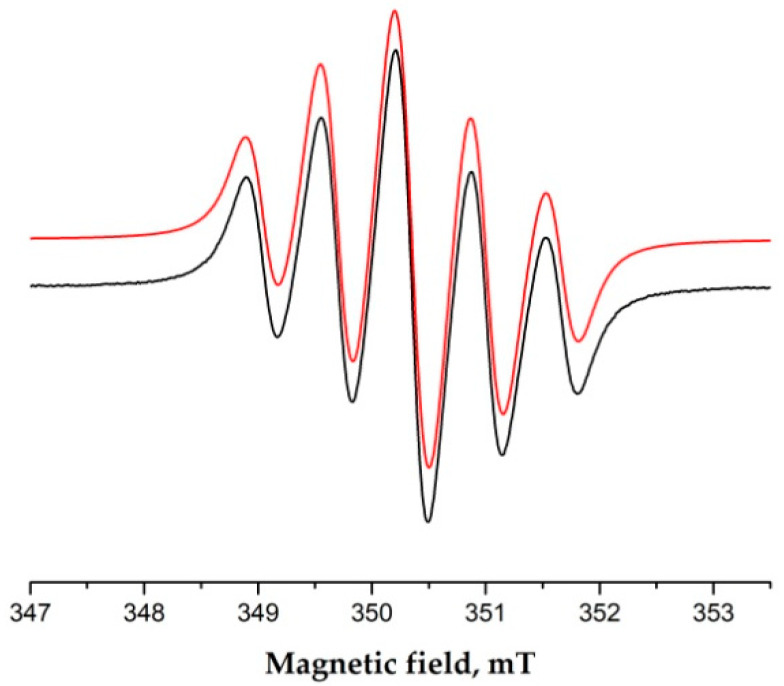
Experimental (black curve) and simulated (red curve) ESR (electron spin resonance) spectra of **3**.

Electron spin resonance (ESR) spectra of diluted (~10^−4^ M) chloroform solutions of diradical **3** showed the quintet with an intensity ratio of 1:2:3:2:1 (Figure 1), which is characteristic of biradicals with a strong exchange interaction. A simulated spectrum (Figure 1, red curve) was calculated with *J* > 1300 MHz and A_N_ = 1.31 mT. Indeed, calculations at the BS-DFT level predicted for the optimized structure of **3** the value of |*J*| ~140 GHz.

**Figure 2:**
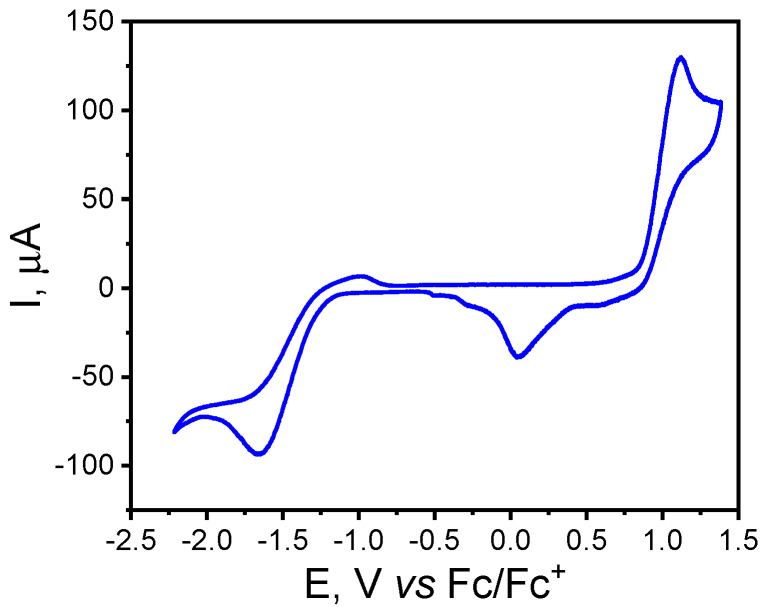
A cyclic voltammogram of *N*,*N*′-(perfluorobiphenyl-4,4′-diyl)bis(*N*-*tert*-butyl(oxyl)amine **3** in a CH_2_Cl_2_ solution.

Electrochemical properties of *N*,*N*′-(perfluorobiphenyl-4,4′-diyl)bis(*N*-*tert*-butyl(oxyl)amine **3** were evaluated by cyclic voltammetry measurements in a CH_2_Cl_2_ solution (Figure 2). Diradical **3** featured an irreversible oxidation wave with E_1/2_ ≈ 0.98 V, which was assigned to the oxidation of the nitroxide radicals into the corresponding oxoammonium cations. On the cathodic side, nitroxide diradical **3** also showed irreversible redox at −1.43 V. The redox potentials of diradical **3** are very close to those of fluorinated phenyl-*tert*-butylnitroxides reported by us previously [25].

**Figure 3:**
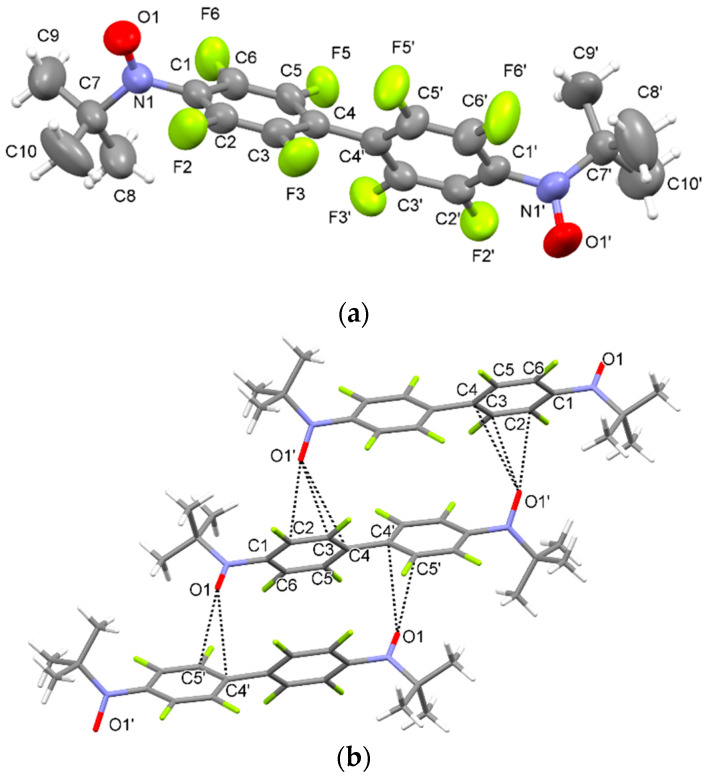
Molecular structure (**a**) and a fragment of crystal structure (**b**) of nitroxide diradical **3** (the thermal ellipsoids are drawn at the 50% probability level).

By crystallization from a cold *n*-heptane solution, we managed to isolate diradical **3** as high-quality crystals and solved its molecular and crystalline structures by X-ray diffraction (XRD) analysis. Diradical **3** crystallizes in the monoclinic *P2_1_*/*c* space group. Bond lengths of the *tert*-butylnitroxide moiety are completely compatible with those of previously described radicals of this family [25]. The nitroxide groups in diradical **3** are twisted by different and large angles (~65° and ~70°) relative to the aromatic rings, as are the aromatic rings toward each other (~53°, Figure 3a). The observed twisting of the diradical is obviously due to the mutual effects of (i) steric repulsion between the *tert*-butyl group and phenylene *ortho*-fluorines, (ii) steric repulsion between the neighboring fluorine atoms of phenylenes, and (iii) electrostatic repulsion of dipoles C–F and N–O. In this regard, a similar dihedral angle in nonfluorinated *tert*-butyl phenyl nitroxides is twofold smaller and has experimental and calculated values of 23–34° [26].

Analysis of crystal packing of nitroxide diradical **3** revealed short contacts O1…C5′ with distance equal to 3.046(4)Å and O1…C4′ with distance equal to 3.217(4) Å, which is, shorter than the sum of van der Waals radii of O and C atoms (3.35 Å) [27]. Additionally, there are N1′–O1′…π(C1–C6) interactions with O…Cg distances of 3.427(4) Å and an O…π-plane distance (Dpln) of 3.358 Å. The above interactions and contacts formally bind molecules into chains along the (*a* + *c*) direction (Figure 3b).

For the crystal structure of diradical **3**, parameters *J* of the intra- and intermolecular exchange interactions were also calculated at the BS-DFT level. Antiferromagnetic (AFM) interaction with *J* = −2.65 cm^−1^ was predicted for the diradical **3**. The AFM interactions were also predicted for the radical fragments of the neighboring diradicals: *J* = −1.14 cm^−1^ for fragments with O1 and O1′ atoms and −1.62 cm^−1^ for fragments with both O1 type atoms (see Figure 3).

We performed also partial optimization of the structure of diradical **3** keeping planar or perpendicular alignment of phenyl rings of the biphenyl bridge. Perpendicular alignment leads to negligible *J* ~ −0.2 cm^−1^, and a very unfavorable planar geometry leads to slightly stronger AFM interaction with *J* ~ −13 cm^−1^.

## 3. Conclusions

We demonstrated that the substitution of fluorine atoms in polyfluorinated arenes with *tert*-butylamine and the oxidation of the resultant substituted diamines with *m*-CPBA can be used to obtain fluorinated multi-spin organic molecules. Via this approach, a new nitroxide diradical, i.e., *N*,*N*′-(perfluorobiphenyl-4,4′-diyl)bis(*N*-*tert*-butyl(oxyl)amine), was successfully prepared from perfluorobiphenyl. For the crystal structure geometry, calculated intra- and intermolecular exchange interactions are well balanced and not so high (*J* from −2.65 to −1.14 cm^−1^). Therefore, this makes this diradical a promising crystalline material to probe a field-induced higher-ordered magnetic state at ultracold temperatures [28]. Apart from this, the synthesized diradical is stable and, according to pleliminary experiments, can be used for preparation of heterospin metal complexes, including those with chain polymer structure.

In general, the proposed approach to multi-spin organic molecules can be used: (i) to rationally design and prepare diradicals with well-balanced sizes of intra- and interdimer exchange couplings for studying of triplet excitations at ultra-cold temperatures, (ii) to provide access to a wide variety of polyfluorinated di- and polyradicals, which need to be synthesized and crystallized to further reveal their inherent magneto-structural correlations, and (iii) to open ways to a practically unknown group of hybrid magnets based on metal complexes with polyfluorinated radicals with enhanced stability and volatility.

## 4. Materials and Methods

### 4.1. Reagents and General Methods

Chemicals were of the highest purity commercially available and were used as received. The progress of reactions was monitored by thin-layer chromatography (TLC) on Silica gel 60 F_254_ aluminum sheets with hexane or CHCl_3_ as the eluent. NMR spectra were recorded on Bruker Avance-300 (300.13 MHz for ^1^H, 282.25 MHz for ^19^F) and Avance-400 (400.13 MHz for ^1^H, 100.62 MHz for ^13^C) spectrometers; chemical shifts (δ) of ^1^H and ^13^C{1H} are given in ppm, with solvent signals serving as the internal standard (δH = 7.24 ppm, δC = 76.9 ppm); the internal standard for ^19^F spectra was C_6_F_6_ (δ = −162.9 ppm). Fourier transform infrared (FT-IR) spectra were acquired in KBr pellets on a Bruker Vector-22 spectrometer. UV-vis spectra were registered on an HP Agilent 8453 spectrophotometer (in 10^−5^–10^−4^ M solutions in EtOH). Masses of molecular ions were determined by high-resolution mass spectrometry (HRMS) by means of a DFS Thermo Scientific instrument (EI, 70 eV). Melting points were recorded on a Melter-Toledo FP81 Thermosystem apparatus.

### 4.2. Synthesis of N4,N4′-di-tert-butyl-2,2′,3,3′,5,5′,6,6′-octafluorobiphenyl-4,4′-diamine 2

A 2.5 M solution of *n*-butyllithium (1.6 mL, 4.0 mmol) in THF was added at −90 °C to a vigorously stirred solution of *tert*-butylamine (292 mg, 4.0 mmol) in THF (20 mL) under argon. The reaction mixture was stirred at −90 °C for 10 min. Then, a solution of perfluorobiphenyl (334 mg, 1.0 mmol) in THF (10 mL) was added, and the reaction mixture was allowed to warm up to room temperature. After 12 h, the stirring was stopped, and the reaction mixture was brought into contact with the air. Flash chromatography (SiO_2_, column 3 × 4 cm, EtOAc as the eluent) yielded a crude product (297 mg). The product was purified by layer chromatography (SiO_2_, hexane as the eluent) to obtain 233 mg (53%) of the title product in the form of colorless crystals. Mp 130–131 °C, *R_f_* = 0.6 (hexane–ethyl acetate 19:1). ^1^H NMR: δ 3.56 (s, 2 H, NH), 1.34 (s, 18 H, *t*-Bu). ^13^C NMR: δ 144.7 (dm, *J_1_* = 251.4 Hz) 139.94 (dm, *J* = 241.5 Hz), 127.36 (t, *J* = 13.8 Hz), 97.57 (tm, *J* = 16.0 Hz), 54.24 (s), 30.34 (t, *J* = 6.0 Hz). ^19^F NMR: δ 20.87 (m, 4 F), 9.48 (d, *J* = 20.9 Hz, 4 F). IR (KBr) 3437, 3396, 3010, 2982, 2939, 2877, 2571, 2434, 2287, 1655, 1606, 1531, 1491, 1468, 1396, 1369, 1288, 1269, 1232, 1203, 1120, 1097, 1041, 974, 926, 887, 818, 733, 714, 656, 633, 575, 494 cm^−1^. HRMS calculated for C_20_H_20_N_2_F_8_ [M]^+^ 440.1493, found 440.1496.

### 4.3. Synthesis of N,N′-(perfluorobiphenyl-4,4′-diyl)bis(N-tert-butyl(oxyl)amine) 3

A solution of diamine **2** (440 mg, 1 mmol) and *m*-CPBA (519 mg, 3.0 mmol) in CHCl_3_ (10 mL) was stirred at room temperature for 12 h. The reaction mixture was filtered through an Al_2_O_3_ layer (column 3 × 20 cm, CHCl_3_ as the eluent). A red fraction of the diradical was collected and evaporated under reduced pressure at room temperature. The residue was dissolved in *n*-heptane (20 mL), and the solution was filtered and kept at −15 °C for 10 h. The crystals were filtered off and air-dried to obtain 360 mg (77%) of the title diradical in the form of red crystals. Mp 132–134 °C (with decomposition), *R_f_* = 0.33 (hexane–ethyl acetate 19:1). IR (KBr) 2985, 2941, 2877, 1790, 1765, 1649, 1606, 1572, 1497, 1477, 1400, 1365, 1350, 1279, 1250, 1200, 1159, 1103, 1003, 976, 849, 789, 733, 719, 581, 474 419 cm^−1^. UV-Vis (EtOH) λ_max_/nm (lg ε): 380 (2.81), 306 (3.76), 237 (4.30), 202 (4.43). HRMS calculated for C_20_H_20_N_2_F_8_O_2_ [M]^+^ 470.1235, found 470.1232.

### 4.4. X-Band ESR Measurements

ESR spectra of **3** were acquired in diluted chloroform solutions at 295 K at concentrations of ~10^−4^ M by means of a commercial Bruker X Band (9 GHz) spectrometer, Elexsys E 540 (Bruker Corporation, Billerica, MA, USA), with the following settings: frequency, 9.87 GHz; microwave power, 2.0 mW; modulation amplitude, 0.05 mT; time constant, 20.5 ms; and conversion time, 20 ms. Simulations of the solution ESR lines were carried out in the EasySpin software, which is available at http://www.easypin.org.

### 4.5. Cyclic Voltammetry Measurements

The electrochemical analysis of **3** was performed in a CH_2_Cl_2_ solution by a computer-controlled P-8 nano potentiostat (Elins, Chernogolovka, Russia) in combination with a three-electrode cell (Gamry, Warminster, PA, USA); 0.1 M tetrabutylammonium hexafluorophosphate served as a supporting electrolyte. Pt, a Pt wire, and Ag/AgCl were used as working, counter, and reference electrodes, respectively. The reference electrode was calibrated by measuring the redox potential of ferrocene. The scan rate was 100 mV/s.

### 4.6. Crystallographic Analysis

XRD data for **3** were collected on a Bruker Kappa Apex II CCD diffractometer (Mo Kα radiation and a graphite monochromator). Absorption corrections were applied empirically using SADABS programs [29]. The structure was solved by direct methods in SHELXS-97 [30] and refined by the full-matrix least-squares method against all *F*^2^ in anisotropic approximation by means of the SHELX-2014/7 software suite [31]. Positions of H atoms were calculated geometrically and refined with the riding model. The analysis of intermolecular contacts was performed in the PLATON software [32,33].

Crystallographic data for **3**: C_20_H_18_N_2_O_2_F_8_, *M* 470.36, monoclinic, *P2_1_/c*, *a* 10.158(2), *b* 19.215(4), *c* 12.002(2) Å, β 114.646(9)°, *V* 2129.2(8) Å^3^, *Z* 4, *D*_calcd_ 1.467 g·cm^−3^, *μ*(Mo-*K*α) 0.142 mm^−1^, F(000) 960, (*θ* 2.15°–26.14°, completeness 99.9%), T 296(2) K, colorless, (0.66 × 0.23 × 0.06) mm^3^, transmission 0.7845–0.8620, 28199 measured reflections in index range −12 ≤ h ≤ 12, −23 ≤ k ≤ 23, −14 ≤ l ≤ 14, 4194 independent (*R*_int_ 0.0331), 295 parameters, *R*_1_ 0.0529 (for 2862 observed *I >* 2*σ*(*I*)), *wR*_2_ 0. 1654 (all data), GOOF 1.033, largest diff. peak and hole 0.272 and −0.215 e·A^−3^.

CCDC 2004458 contains the Supplementary Materials crystallographic data for this paper. These data can be obtained free of charge via http://www.ccdc.cam.ac.uk/cgi-bin/catreq.cgi or from the Cambridge Crystallographic Data Centre, 12 Union Road, Cambridge, CB2 1EZ, UK; fax: (+44) 1223 336 033 or e-mail: deposit@ccdc.cam.ac.uk.

### 4.7. Computational Details

Parameters of the intra- and intermolecular exchange interactions (*J*) were computed for pairs of the radical centers ($\hat{H}=-2J{\hat{\vec{S}}}_{1}{\hat{\vec{S}}}_{2}$) using the spin-unrestricted broken-symmetry (BS) approach [34]. The energies of the high-spin state ($E^{HS}$) and low-spin state within the BS approach ($E_{BS}^{LS}$) were calculated at the UB3LYP/def2-TZVP level of theory [35,36,37], and the *J* values were computed according to the following formula [38]:

(1) $$J=-\frac{E^{HS}-E_{BS}^{LS}}{\langle S^{2}\rangle^{HS}-\langle S^{2}\rangle_{BS}^{LS}}.$$

The XRD or UB3LYP/def2-TZVP optimized geometries of diradical **3** were used in the calculations. Pairs of radical fragments of neighboring diradicals were obtained from the XRD structure of the diradical pair by replacing the corresponding NO groups by NOH groups. All calculations were performed using the ORCA 4.0.1 software package [39].

## Supplementary Materials

The following is available online: NMR and IR spectra of **2** and IR and UV/Vis spectra of **3**.
