# Supplementary material for: Synthesis of Nitroxide Diradical Using a New Approach
Source: Molecules. 2020 Jun 11;25(11):2701. doi: 10.3390/molecules25112701 (PMC7321179; doi:10.3390/molecules25112701)
Supplement: Supplementary file 1 [file molecules-25-02701-s001.pdf]

molecules

Supplementary Materials

Synthesis of Nitroxide Diradical Using a New Approach

Pavel Fedyushin [1], Tatyana Rybalova [1], Nargiz Asanbaeva [1,2], Elena Bagryanskaya [1,2], Alexey Dmitriev [2,3], Nina Gritsan [2,3], Maxim Kazantsev [1,4] and Evgeny Tretyakov [1,4,*]

1 N. N. Vorozhtsov Institute of Organic Chemistry, 9 Ac. Lavrentieva Avenue, 630090 Novosibirsk, Russia; feduyshin@nioch.nsc.ru (P.F.); rybalova@nioch.nsc.ru (T.R.); nasanbaeva@nioch.nsc.ru (N.A.); egbagryanskaya@nioch.nsc.ru (E.B.); maximkazantsev1988@gmail.com (M.K.)

2 Department of Physics, Novosibirsk State University, 2 Pirogova Str., 630090 Novosibirsk, Russia; dmitralexey@gmail.com (A.D.); nina.gritsan@gmail.com (N.G.)

3 V. V. Voevodski Institute of Chemical Kinetics and Combustion, 3 Institutskaya Str., 630090 Novosibirsk, Russia

4 Department of Natural Sciences, Novosibirsk State University, 2 Pirogova Str., 630090 Novosibirsk, Russia;

* Correspondence: tretyakov@nioch.nsc.ru

Table of contents:

NMR spectra of diamine **2**……………………………………………………S2-S4
IR spectrum of diamine **2**……………………………………………………S5
IR spectrum of diradical **3**……………………………………………………S6
UV/Vis spectrum of **3**…………………………………………………………S7

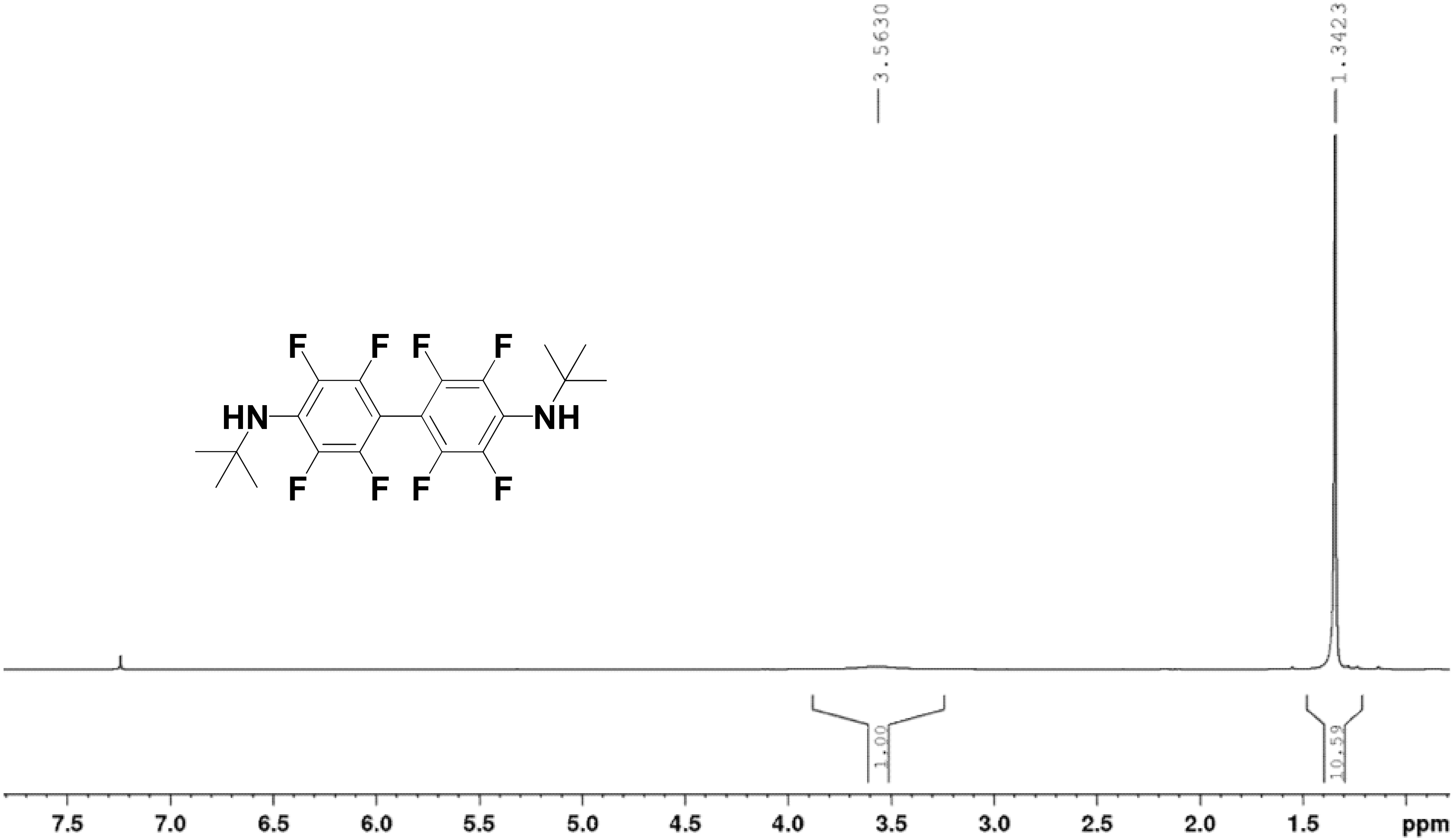

3.5630
1.3423
HN
NH
F
1.00
10.59
7.5
7.0
6.5
6.0
5.5
5.0
4.5
4.0
3.5
3.0
2.5
2.0
1.5
ppm

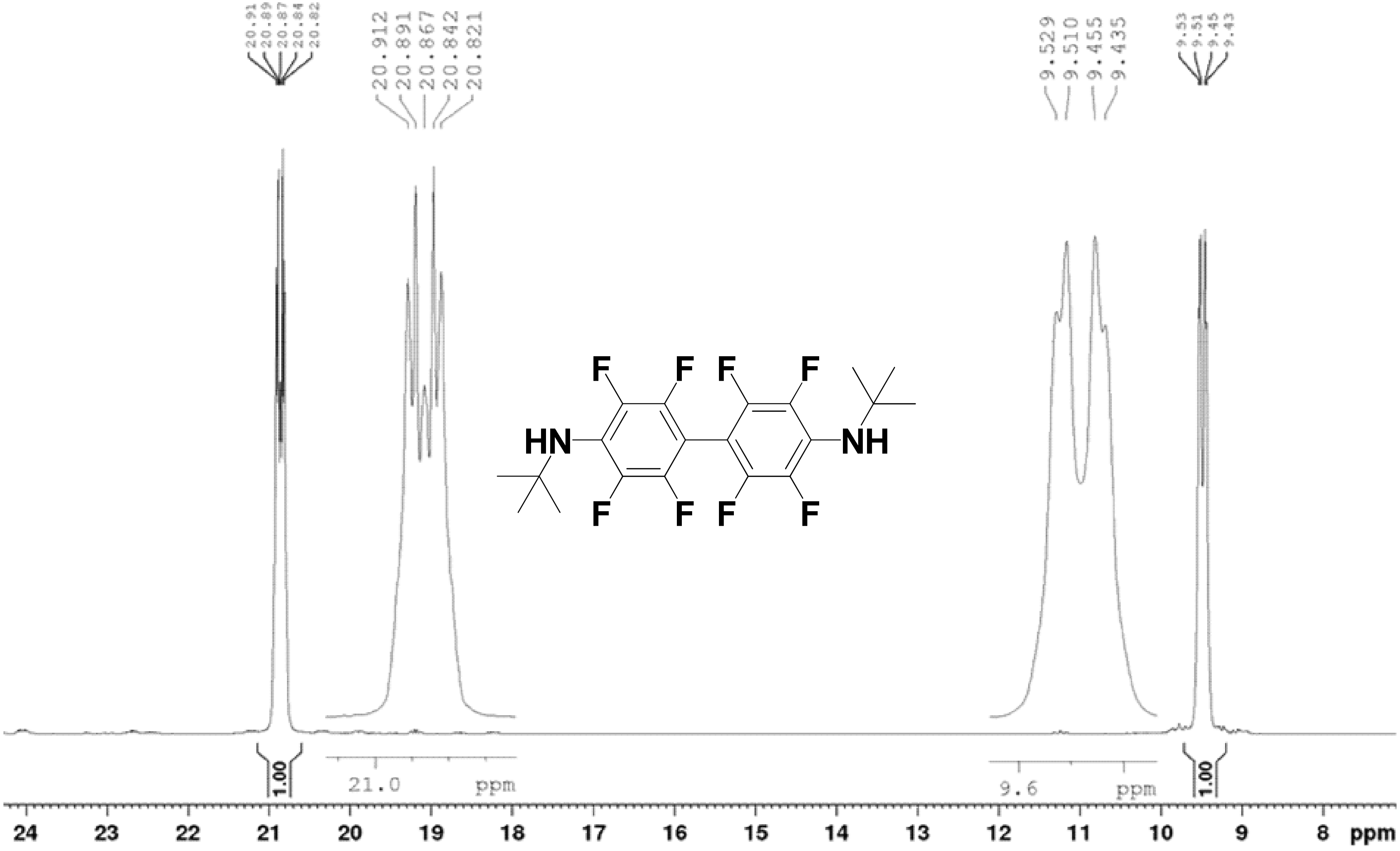
20.91
20.89
20.87
20.84
20.82
20.912
20.891
20.867
20.842
20.821
9.529
9.510
9.455
9.435
9.53
9.51
9.45
9.43
F
F
F
F
HN
NH
F
F
F
F
1.00
21.0
ppm
9.6
ppm
1.00
24
23
22
21
20
19
18
17
16
15
14
13
12
11
10
9
8
ppm

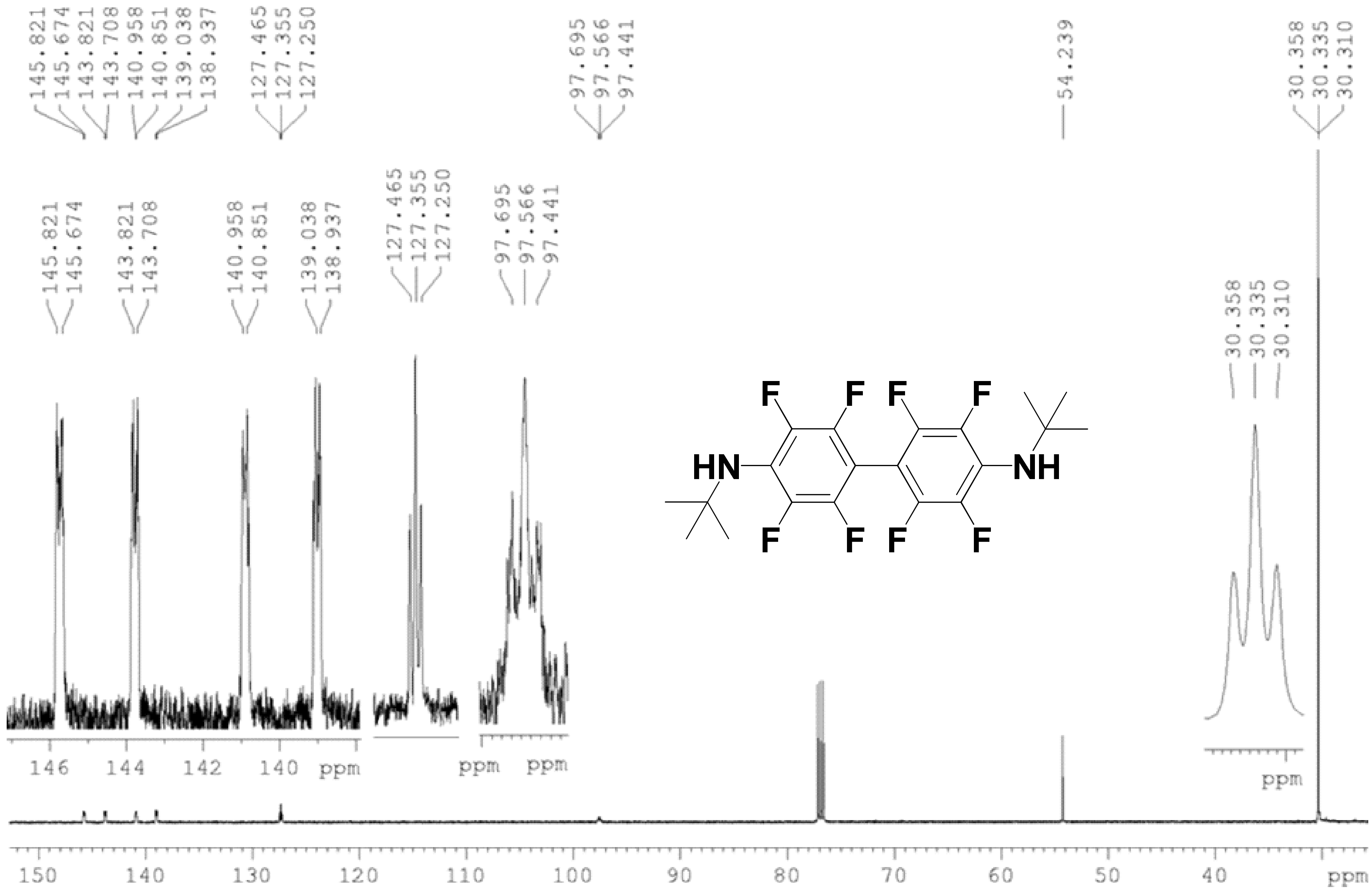
145.821
145.674
143.821
143.708
140.958
140.851
139.038
138.937
127.465
127.355
127.250
97.695
97.566
97.441
54.239
30.358
30.335
30.310
146
144
142
140
ppm
ppm
ppm
ppm
F
F
F
F
HN
NH
F
F
F
F
150
140
130
120
110
100
90
80
70
60
50
40
ppm

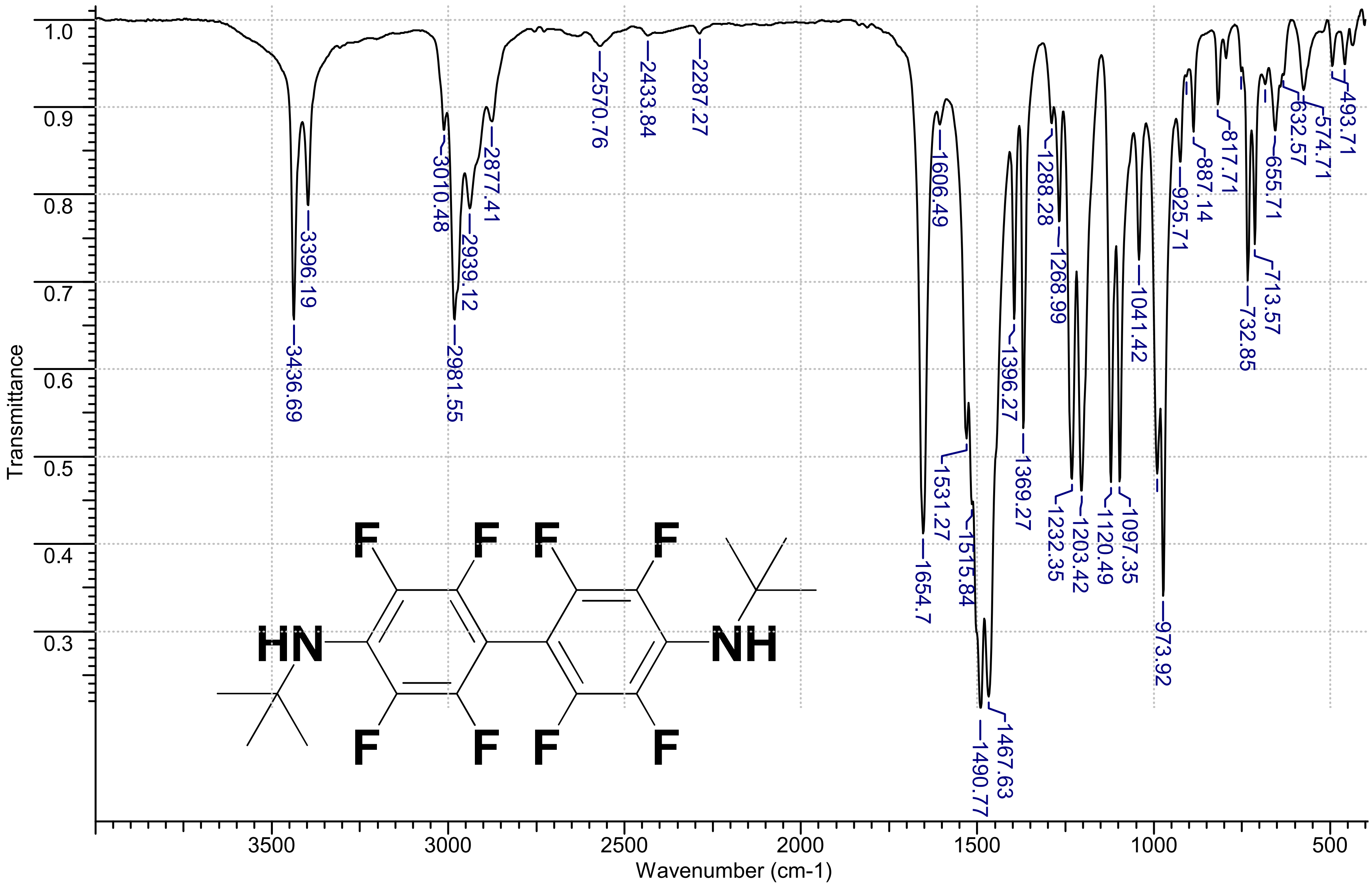
Transmittance
Wavenumber (cm-1)
1.0
0.9
0.8
0.7
0.6
0.5
0.4
0.3
3500
3000
2500
2000
1500
1000
500
3436.69
3396.19
3010.48
2981.55
2939.12
2877.41
2570.76
2433.84
2287.27
1654.7
1606.49
1531.27
1515.84
1490.77
1467.63
1396.27
1369.27
1288.28
1268.99
1232.35
1203.42
1120.49
1097.35
1041.42
973.92
925.71
887.14
817.71
732.85
713.57
655.71
632.57
574.71
493.71
HN
NH
F

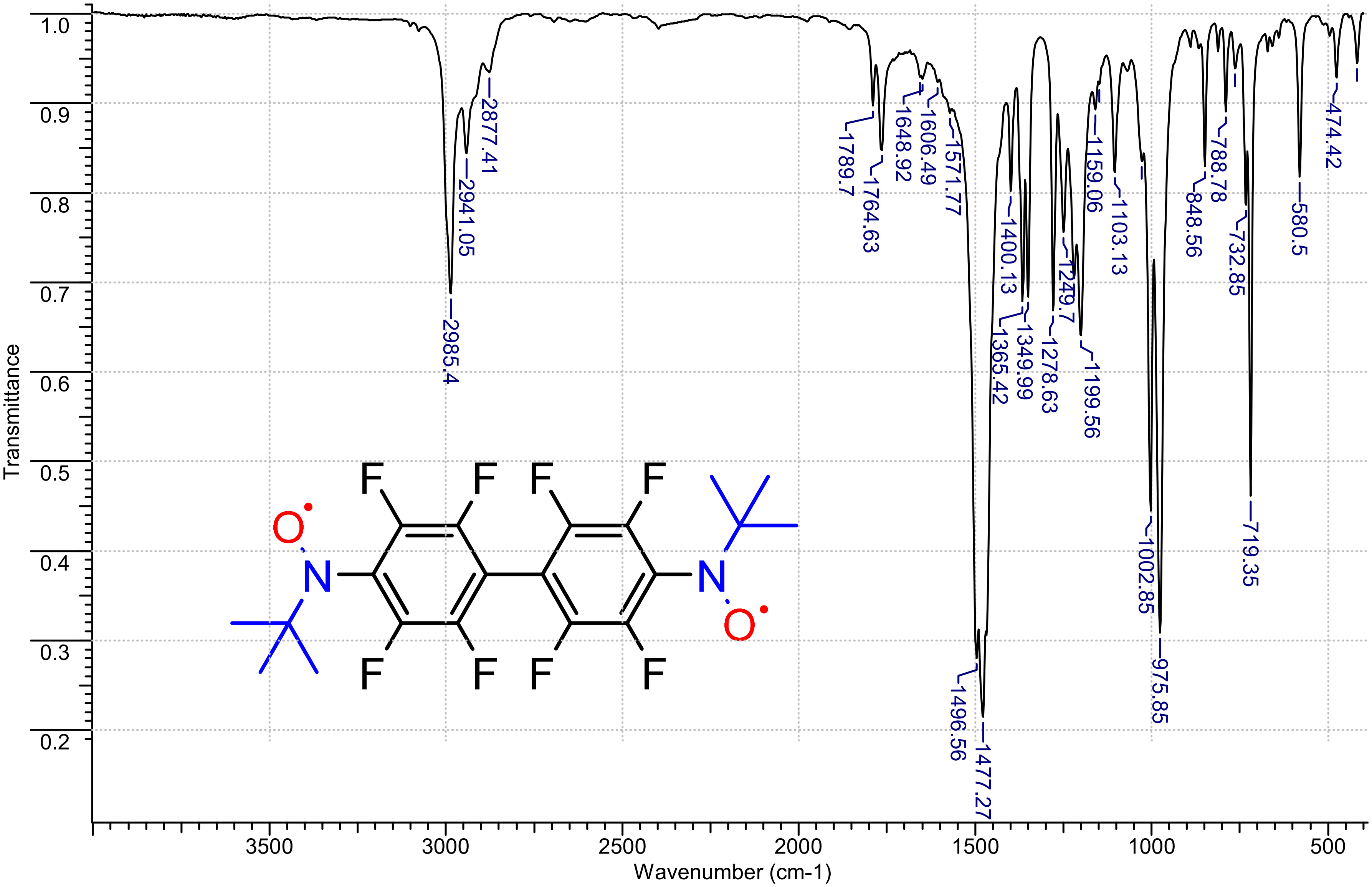
Transmittance
1.0
0.9
0.8
0.7
0.6
0.5
0.4
0.3
0.2
3500
3000
2500
2000
1500
1000
500
Wavenumber (cm-1)
2985.4
2941.05
2877.41
1789.7
1764.63
1648.92
1606.49
1571.77
1496.56
1477.27
1400.13
1365.42
1349.99
1278.63
1249.7
1199.56
1159.06
1103.13
1002.85
975.85
848.56
788.78
732.85
719.35
580.5
474.42
F
N
O

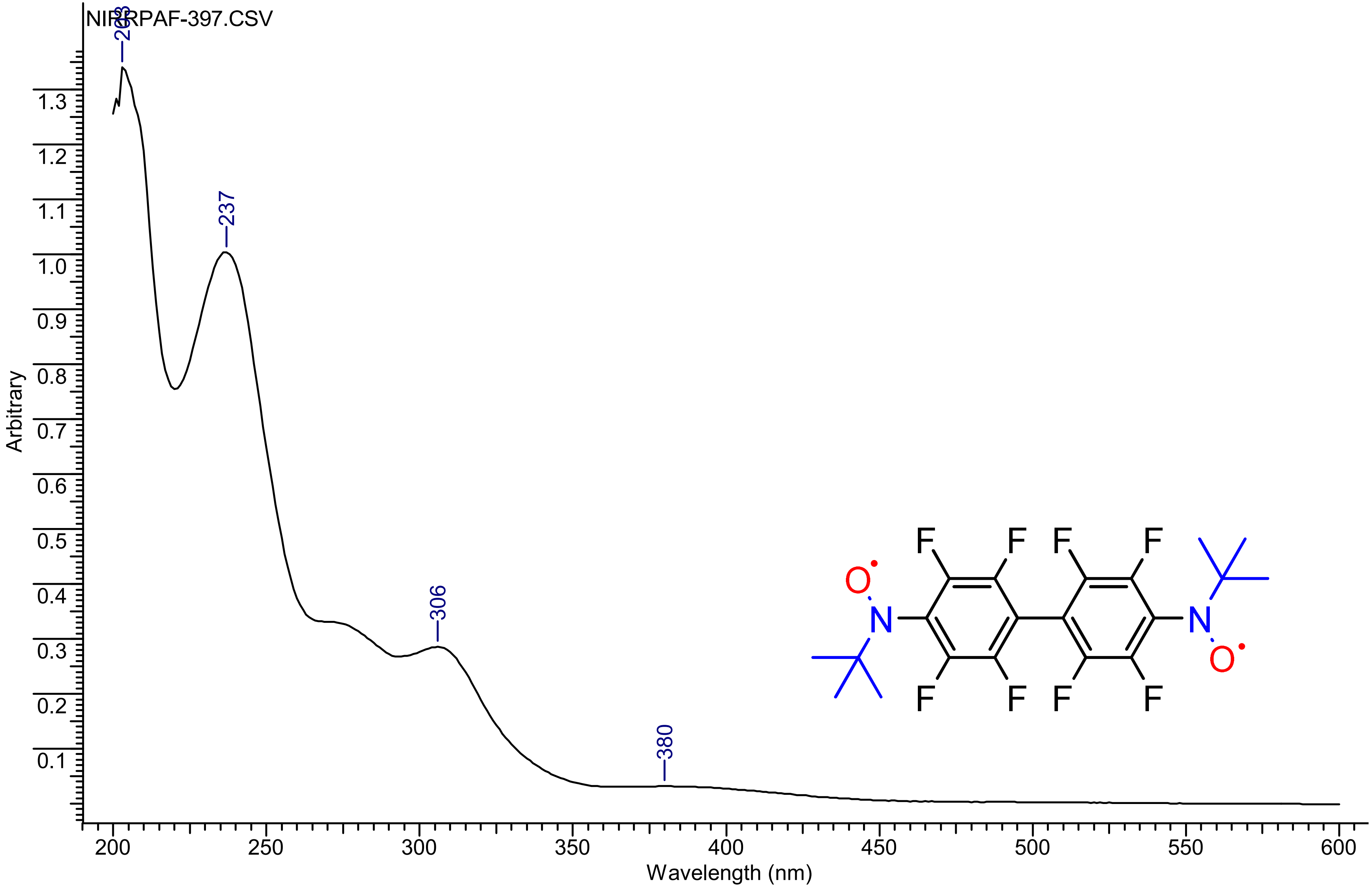

NIRRPAF-397.CSV
203
237
306
380
1.3
1.2
1.1
1.0
0.9
0.8
0.7
0.6
0.5
0.4
0.3
0.2
0.1
Arbitrary
200
250
300
350
400
450
500
550
600
Wavelength (nm)
